# Congenital pleuropulmonary blastoma in a newborn with a variant of uncertain significance in *DICER1* evaluated by RNA-sequencing

**DOI:** 10.1186/s40748-023-00148-2

**Published:** 2023-03-16

**Authors:** Allison N. J. Lyle, Timothy J. D. Ohlsen, Danny E. Miller, Gabrielle Brown, Natalie Waligorski, Rebecca Stark, Mallory R. Taylor, Mihai Puia-Dumitrescu

**Affiliations:** 1 Department of Pediatrics, Division of Neonatal-Perinatal Medicine, University of Washington, Seattle Children’s Hospital, 4800 Sand Point Way NE, 98105 Seattle, WA USA; 2 Department of Pediatrics, Division of Pediatric Hematology/Oncology, University of Washington, Seattle Children’s Hospital, 4800 Sand Point Way NE, WA 98105 Seattle, USA; 3 Department of Pediatrics, Division of Genetic Medicine, Department of Laboratory Medicine & Pathology, University of Washington, Seattle Children’s Hospital, 4800 Sand Point Way NE, 98105 Seattle, WA USA; 4 Department of Pediatrics, Division of Genetic Medicine, University of Washington, Seattle Children’s Hospital, 4800 Sand Point Way NE, 98105 Seattle, WA USA; 5 Department of Surgery, Division of Pediatric General and Thoracic Surgery, University of Washington, 4800 Sand Point Way NE, 98105 Seattle, WA USA

**Keywords:** Neonatal intensive care unit, pleuropulmonary blastoma, congenital pulmonary airway malformation, *DICER1*, *DICER1* syndrome, RNA sequencing

## Abstract

**Background:**

Pleuropulmonary blastoma (PPB) is a rare mesenchymal malignancy of the lung and is the most common pulmonary malignancy in infants and children. Cystic PPB, the earliest form of PPB occurring from birth to approximately two years of age, is often mistaken for a congenital pulmonary airway malformation, as the two entities can be difficult to distinguish on imaging and pathology. Diagnosis of PPB should prompt workup for *DICER1* syndrome, an autosomal dominant tumor predisposition syndrome. We report a newborn with a congenital PPB presenting with tachypnea and hypoxia, who was found to have variant of uncertain clinical significance (VUS) in *DICER1*.

**Case presentation:**

A term female infant developed respiratory distress shortly after birth. Initial imaging was concerning for a congenital pulmonary airway malformation versus congenital diaphragmatic hernia, and she was transferred to a quaternary neonatal intensive care unit for management and workup. Chest CT angiography demonstrated a macrocytic multicystic lesion within the right lower lobe without systemic arterial supply. The pediatric surgery team was consulted, and the neonate underwent right lower lobectomy. Pathology revealed a type I PPB. Oncology and genetics consultants recommended observation without chemotherapy and single gene sequencing of *DICER1*, which identified a germline VUS in *DICER1* predicted to alter splicing. RNA-sequencing from blood demonstrated that the variant resulted in an in-frame deletion of 29 amino acids in a majority of transcripts from the affected allele. Due to the patient’s young age at presentation and high clinical suspicion for *DICER1* syndrome, tumor surveillance was initiated. Renal and pelvic ultrasonography were unremarkable.

**Conclusion:**

We present the case of a term neonate with respiratory distress and cystic lung mass, found to have a type I PPB with a germline VUS in *DICER1* that likely increased her risk of *DICER1*-related tumors. Nearly 70% of patients with PPB demonstrate germline mutations in *DICER1*. Review of RNA sequencing data demonstrates the difficulty in classifying splice variants such as this. Penetrance is low, and many patients with pathogenic *DICER1* variants do not develop a malignancy. Best practice surgical and oncologic recommendations include an individualized approach and tumor board discussion. This case highlights the importance of a multidisciplinary team approach and the utility of international registries for patients with rare diagnoses.

## Background

Pleuropulmonary blastoma (PPB) is a rare mesenchymal malignancy of the lung, with an incidence of 25–50 cases per year in the United States [1, 2]. Cystic PPB, the earliest form of PPB occurring from birth to approximately two years of age, [3–5] is often mistaken for a congenital pulmonary airway malformation (CPAM), as the two entities can be difficult to distinguish on imaging and pathology [6–8]. Diagnosis of PPB should prompt workup for *DICER1*syndrome, a cancer predisposition syndrome strongly associated with PPB [9]. We report a newborn with a congenital PPB presenting with tachypnea and hypoxia, who was found to have a variant of uncertain clinical significance (VUS) in *DICER1* predicted to alter mRNA splicing. RNA-sequencing of blood-derived RNA confirmed that the variant resulted in a 29-amino acid in-frame deletion in a minority of transcripts, suggesting possible increased risk for the development of PPB and other *DICER1*-related tumors. This orthogonal data allowed us to reclassify the variant as likely pathogenic using *DICER1*-specific classification criteria [10, 11]. Together, the patient’s phenotype and laboratory findings are highly suspicious for *DICER1* syndrome. This case represents a PPB diagnosis at a very early age and demonstrates how RNA-sequencing might be used to clarify a VUS identified by clinical genetic testing.

## Case presentation

**Fig. 1:**
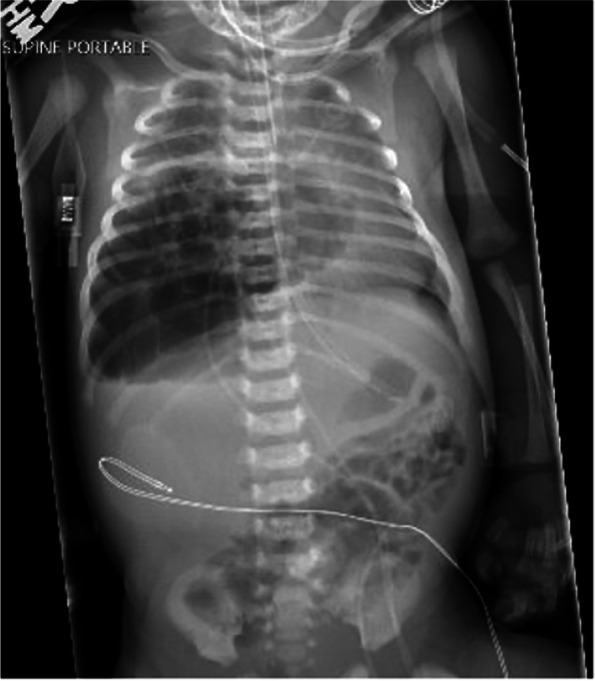
Initial chest radiograph demonstrates a possible congenital diaphragmatic hernia versus multicystic mass causing a left shift

**Fig. 2:**
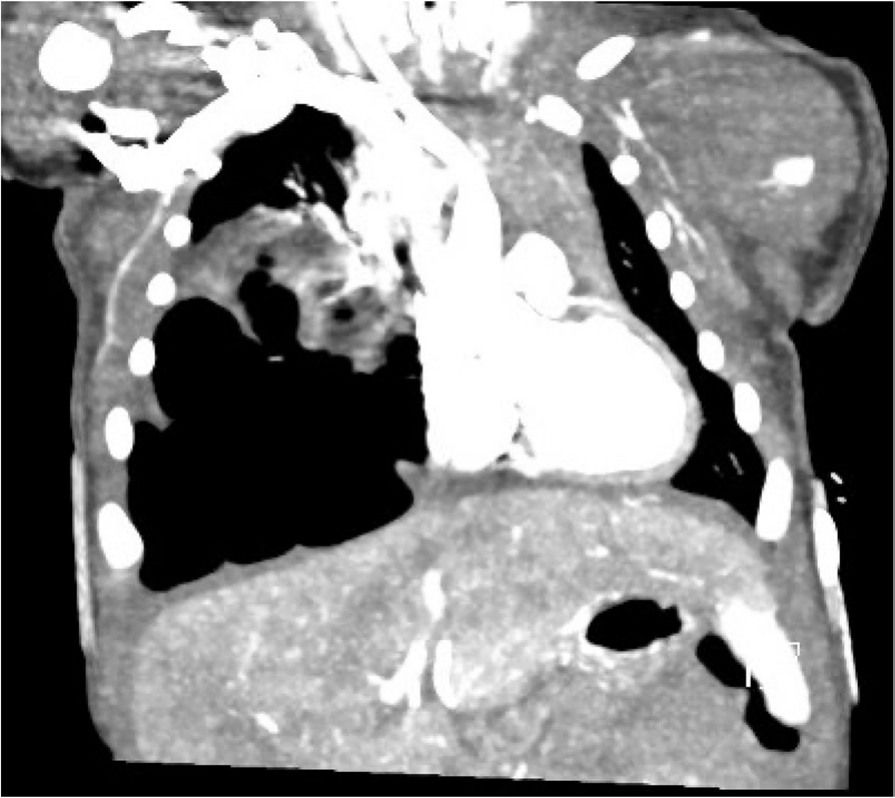
Chest computed tomography angiogram demonstrates a macrocystic multicystic lesion within the right lower lobe without systemic arterial supply

A 3.055-kg term female infant was born via Cesarean section due to failure to progress after induction of labor for gestational hypertension following an otherwise uncomplicated pregnancy. No resuscitation was required at birth and APGAR scores were 8 and 9 at 1 and 5 min, respectively. At 14 h of life, the neonate developed respiratory distress and hypoxia following breastfeeding, necessitating transfer to the neonatal intensive care unit (NICU) where she was placed on 6 L high flow nasal cannula and a maximum FiO2 of 0.26. The initial exam was significant for diminished breath sounds on the right chest, intermittent tachypnea, and bilateral mild subcostal retractions. Chest x-ray obtained on admission to the NICU was concerning for a possible congenital diaphragmatic hernia (CDH) versus multicystic mass causing a left shift (Fig. 1). An ultrasound of the lung demonstrated a right multicystic mass at the base concerning for CPAM and an intact diaphragm. The neonate was transported to a Level IV NICU for further multidisciplinary evaluation and management. Repeat chest x-ray was read as a right CDH versus CPAM filling much of the right chest with leftward mediastinal shift. Chest CT angiography demonstrated a macrocytic multicystic lesion within the right lower lobe without systemic arterial supply (Fig. 2). The pediatric surgery team was consulted, and the neonate underwent right lower lobectomy on day of life 4.

Pathology revealed a type I PPB. The oncology and genetics teams were consulted and recommended close observation without chemotherapy, as well as single gene sequencing of *DICER1* because of concern for *DICER1* syndrome. Concurrently, due to the patient’s very young age at presentation and high clinical suspicion for *DICER1* syndrome, tumor surveillance was initiated while genetic testing was pending. Renal and pelvic ultrasonography were unremarkable. The neonate’s course was complicated by a right-sided pneumothorax necessitating chest tube placement; this resolved, and she was discharged home with the family on day of life 43 on room air with close follow-up with oncology, surgery, and pulmonology.

Peripheral blood sequencing of the *DICER1* gene identified a heterozygous VUS (NM_177438.2:c.2523 A > G, p.Q841Q) that was absent from population databases and had not been previously reported. Computational tools [12–14] predicted that this synonymous variant would create a new splice acceptor site in exon 16 of *DICER1*leading to an in-frame deletion of 29 amino acids of exon 16 between the new acceptor site and the original acceptor site at the beginning of the exon. This exon is highly conserved through at least Zebrafish and the deleted region is predicted to form secondary structures including alpha and beta structures. Individuals with PPB and smaller deletions within this region have been reported [15]. Unfortunately, using computational tools alone, it was not possible to determine the frequency of aberrant transcript formation, limiting the interpretation of the variant’s clinical impact.

Paired tumor-peripheral blood sequencing of *DICER1*was sent and confirmed the VUS was present in the tumor [16]. In addition, the tumor was positive for a known pathogenic *DICER1*RNase IIIb domain mutation (c.5125G > A, p.D1709N) [17]. Clinical RNA sequencing was then performed to determine the impact of the VUS on splicing or expression. The clinical report stated that a minority of transcripts utilized the novel AG acceptor site resulting in an in-frame deletion of the first 29 amino acids of exon 16 and was interpreted as an indeterminate result by the clinical laboratory. No other splicing abnormalities were identified, and the clinical lab reported that *DICER1*expression was similar to tissue-matched controls. Thus, the c.2523 A > G variant remained a VUS. Review of the raw sequencing data obtained from the clinical testing lab revealed that 3/61 transcripts contained the c.2523 A > G variant (5% of all transcripts) and 9 of 70 mapped transcripts contained the 29 amino acid deletion. Thus, transcripts carrying the c.2523 A > G variant appeared to be underrepresented in the sequencing data. Furthermore, a single nucleotide polymorphism in the 3’UTR of DICER1 (g.14:95554142T > C) was observed in the RNA sequencing data at an allele frequency of 53% (present in 50 of 95 total mapped reads), suggesting that transcription of both alleles of DICER1 is occurring and are represented equally in the data. Thus, we were able to reclassify the variant as likely pathogenic using the variant interpretation guidelines (version 1.1.0) for DICER 1 developed by the ClinGen DICER1 and miRNA-processing gene expert panel using criteria PS3 (moderate), PS4 (supporting), PM2 (supporting), PP3 (supporting), and PP4 (supporting) which resulted in a modified Bayesian point score of 6 [10, 18].

The infant was enrolled on the International PPB Registry [19] and followed up with the oncology and pediatric surgery teams shortly after discharge. Given the young age of the patient, an observation-only approach, as opposed to adjuvant chemotherapy, was taken. CT chest imaging at 1-, 4-, 8-, and 14-months post-surgery did not identify recurrent tumor. The patient is now nearly 18 months post-surgery and is growing well with appropriate development. She exhibits no symptoms of respiratory distress. She will continue to receive surveillance for PPB recurrence or the development of additional neoplasms related to her presumed *DICER1* syndrome. The patient’s parents were offered genetic testing for themselves but declined, therefore it is not certain whether this variant is *de novo* or inherited.

## **Discussion and conclusion**

We present the case of a term neonate with respiratory distress, found to have a type I PPB and a germline VUS in *DICER1* predicted to affect splicing. *DICER1* is a member of the ribonuclease II family and is involved in the generation of microRNAs that modulate posttranscriptional gene expression. It is involved in many processes, including lung development, maintenance of stem cells, cell cycle progression, and tumorigenesis [20]. Individuals with pathogenic variants in DICER1 are at increased risk of certain malignancies including PPBs, cystic nephromas, ovarian Sertoli-Leydig Cell tumors, genitourinary sarcomas, neuroendocrine tumors, and several other sarcomas [7, 21]. Approximately 70% of all patients with PPB harbor germline mutations in DICER1 that can be identified with standard clinical testing [6–8]. More than 90% of PPBs have at least one somatic or germline pathogenic variant, making genetic sequencing of the tumor very informative [8, 9, 21]. Conversely, penetrance is relatively low, meaning many patients with pathogenic *DICER1*variants do not develop a malignancy during their lifetime. Recent estimates suggest that 5.3% of non-proband DICER1 carriers manifest a phenotype of DICER1 syndrome, not all of which are malignant, by the age of 10, increasing to 19% by age 50 [9]. Screening recommendations include surveillance for the broad range of associated tumors, with particular attention to the chest, abdomen/pelvis, and thyroid [7]. For patients with a diagnosed malignancy, additional surveillance is required.

Sequencing of *DICER1* in the patient demonstrated a germline single nucleotide variant that did not change the amnio acid but was predicted by computational tools to alter splicing which was classified as a VUS. Sequencing of tumor tissue identified a known pathogenic variant in the RNase IIIb domain in addition to the germline VUS, likely representing a second hit. Clinical RNA-sequencing confirmed that the variant did indeed result in a 29-amino acid in-frame deletion in a majority of transcripts; however, because the clinical laboratory felt overall expression of *DICER1*was unchanged the result was classified as indeterminate. Review of the raw RNA sequencing data obtained from the clinical testing lab revealed that 3/61 transcripts contained the c.2523 A > G variant and 9 transcripts with aberrant splicing were identified. Thus, while interpreted as indeterminate by the clinical lab, the fact that only 5% of transcripts contained the c.2523 A > G variant, and the allele frequency of a 3’UTR variant was nearly 50% suggests that a majority of transcripts from that allele are aberrantly spliced. This highlights a challenge with clinical interpretation of RNA-seq data, the benefit of analyzing original sequencing data in uncertain cases, and the need for new technologies, such as long-read RNA sequencing which can capture full isoforms and simplify variant interpretation [22].

In summary, we present the case of a term neonate with a congenital type 1 PPB after presenting with respiratory distress, found to have a germline *DICER1* VUS with altered transcriptional activity by RNA-seq. This case represents the earliest and most curable manifestations of PPB. PPB is a rare mesenchymal malignancy of the lung which may be mistaken for a CPAM due to similarities to PPB in both imaging and pathology. A diagnosis of PPB should always raise suspicion for *DICER1* syndrome, and testing for germline mutations is warranted for patients, and, if positive, their first-degree relatives. *DICER1* syndrome puts individuals at higher risk for other malignancies and a tumor surveillance protocol may be initiated. RNA sequencing is an emerging modality that may clarify VUSs identified by germline testing, but challenges with interpretation and varying practices among clinical laboratories may warrant reanalysis of original sequencing data to fully understand the impact of the genomic change under investigation.

## Data Availability

Not applicable.

## Abbreviations

CDH: Congenital diaphragmatic hernia
CPAM: Congenital pulmonary airway malformation
NICU: Neonatal intensive care unit
PPB: Pleuropulmonary blastoma
VUS: Variant of uncertain significance
