# Correction to: Congenital pleuropulmonary blastoma in a newborn with a variant of uncertain significance in *DICER1* evaluated by RNA-sequencing

**DOI:** 10.1186/s40748-023-00161-5

**Published:** 2023-05-25

**Authors:** Allison N. J. Lyle, Timothy J. D. Ohlsen, Danny E. Miller, Gabrielle Brown, Natalie Waligorski, Rebecca Stark, Mallory R. Taylor, Mihai Puia‑Dumitrescu

**Affiliations:** 1 Department of Pediatrics, Division of Neonatal-Perinatal Medicine, University of Washington, Seattle Children’s Hospital, 4800 Sand Point Way NE, Seattle, WA 98105 USA; 2 Department of Pediatrics, Division of Pediatric Hematology/Oncology, University of Washington, Seattle Children’s Hospital, 4800 Sand Point Way NE, Seattle, WA 98105 USA; 3 Department of Pediatrics, Division of Genetic Medicine, Department of Laboratory Medicine & Pathology, University of Washington, Seattle Children’s Hospital, 4800 Sand Point Way NE, Seattle, WA 98105 USA; 4 Department of Pediatrics, Division of Genetic Medicine, University of Washington, Seattle Children’s Hospital, 4800 Sand Point Way NE, Seattle, WA 98105 USA; 5 Department of Surgery, Division of Pediatric General and Thoracic Surgery, University of Washington, 4800 Sand Point Way NE, Seattle, WA 98105 USA

**Correction: matern health, neonatol and perinatol 9, 4 (2023)**

10.1186/s40748-023-00148-2

Following publication of the original article [1], it was noticed that Gabrielle Brown’s name was spelled incorrectly due to a typesetting error.

Gabrielle Brown’s name is spelled correctly in this erratum, and the original article has been corrected.
